# Altered amplitude of low‐frequency fluctuation in basal ganglia correlates to pulmonary ventilation function in COPD patients: A resting‐state fMRI study

**DOI:** 10.1002/brb3.1336

**Published:** 2019-05-29

**Authors:** Chun‐Qiang Lu, Weiwei Xu, Chu‐Hui Zeng, Lu‐Yao Ge, Yuan‐Cheng Wang, Xiang‐Pan Meng, Qian Yu, Di Wu, Shenghong Ju

**Affiliations:** ^1^ Jiangsu Key Laboratory of Molecular and Functional Imaging, Department of Radiology Zhongda Hospital, Medical School of Southeast University Nanjing China; ^2^ Department of Respirology Zhongda Hospital, Medical School of Southeast University Nanjing China

**Keywords:** ALFF, Basal ganglia, brain, cognitive impairment, COPD, rs‐fMRI

## Abstract

**Introduction:**

Patients under chronic obstructive pulmonary disease (COPD) has been reported to be associated with a higher prevalence of cognitive impairment (CI). However, it is still largely unknown whether the aberrant resting‐state spontaneous neuronal activity pattern reflected by the amplitude of low‐frequency fluctuation (ALFF) analysis will be associated with the CI in COPD patients.

**Materials:**

A total of 28 COPD patients and 26 healthy controls were enrolled in this study. Of all the subjects, structural and functional MRI data, spirometry tests performance and neuropsychological assessments of different cognitive domains were collected. Voxel‐based two‐sample *t* tests were used to detect brain regions showing differences in the ALFF value between COPD patients and healthy controls. An additional fMRI runs with supplementary oxygen delivery were employed to explore the impact of elevated partial pressure of oxygen (PaO_2_) or moderate hyperoxia on ALFF in COPD patients and healthy controls respectively.

**Results:**

More extensive white matter lesion was detected in COPD patients. COPD patients exhibit decreased ALFF value in bilateral basal ganglia areas and right thalamus, and aberrant ALFF value is correlated with PaO_2_ and pulmonary ventilation function (FEV1%pred). COPD patients performed worse in the Digit Span Test (reverse), Digit Symbol Substitution Test, Trail‐making test (A and B) than controls. After supplementary oxygen inhalation, the ALFF value of basal ganglia and right thalamus significantly increased in the controls, but not in the COPD patients.

**Conclusions:**

COPD patients mainly exhibit impaired executive function but not long‐term memory in cognitive function assessment. Aberrant ALFF alteration in the deep brain may be directly related to lower PaO_2_ in COPD patients.

## 1 INTRODUCTION

Chronic obstructive pulmonary disease (COPD) is a common chronic lung disease characterized by incompletely reversible airflow limitation, with shortness of breath and productive cough as main symptoms. There is an increasing recognition that COPD is more than a simple respiratory disorder and associated with many social and psychological problems which include depression, anxiety, and cognitive impairment (CI; Dodd, Getov, & Jones, 2010; Eisner et al., 2011; Fried, Vaz Fragoso, & Rabow, 2012). CI can lead to problems with adherence to treatment, self‐management, and educational achievement (Cleutjens, Spruit, et al., 2017; Sulaiman et al., 2017). Meanwhile, impaired performance in neuropsychological tests had been suggested to be a predictor of mortality and disability (Antonelli‐Incalzi et al., 2006). According to the report of Chang et al., COPD patients with CI had a rate of death nearly three times as high as the sum of COPD patients without CI and patients only had CI in their hospitalization (Chang, Chen, McAvay, & Tinetti, 2012).

The pathogenesis of CI in COPD patients is largely unknown. Possible mechanisms contributing to CI in COPD include inflammation and small vascular disease mediated through chronic hypoxia, heavy metals, nicotine accumulation caused by cigarette smoke, and other comorbidities (Dodd et al., 2010; Lahousse, Tiemeier, Ikram, & Brusselle, 2015). Different pathways in COPD pathogenesis may affect different brain regions associated with different brain functions and may lead to a pattern of cognitive dysfunction specific to COPD. There have been some studies concerning the subtypes of MCI (mild cognitive impairment) or specific affected cognitive domains in the COPD patients (Cleutjens, Franssen, et al., 2017; Singh et al., 2014, 2013; Villeneuve et al., 2012). Most of these studies indicate that COPD patients with an increased risk of na‐MCI (non‐amnestic MCI), while an increased risk of a‐MCI (amnestic MCI) has also been reported. Thus, it is still of importance to investigate the affected domains of cognitive function specific to COPD for a better understanding of the pattern of CI in COPD.

Meanwhile, a few studies have attempted to explore the brain pathology involving the brain structural change and vascular disease in the COPD patient through MRI (Cleutjens, Ponds, et al., 2017; Wang et al., 2017). However, the number of such studies is still small and the conclusions are lack of agreement. Studies incorporating structural and functional MRI can provide more comprehensive information for the underlying mechanism of the various pathways in the pathogenesis of CI (Dodd et al., 2012). Chronic hypoxia is supposed to be the most important part for the neuronal damage through inflammation and atherosclerosis in the COPD (Dodd et al., 2010). Spontaneous or task‐related neuronal activity is also reported to be suppressed under both experimental and chronic hypoxia environment (Gavello et al., 2012; Goodall, Twomey, & Amann, 2014; Sicard & Duong, 2005; Sumiyoshi, Suzuki, Shimokawa, & Kawashima, 2012). Amplitude of low‐frequency fluctuation (ALFF) is a resting‐state functional MRI (rs‐fMRI) method which may serve as a surrogate for neural activity at single‐voxel level (Cui et al., 2014; Zang et al., 2007). Given that, an analysis of ALFF may provide important information for the spontaneous neuronal activity pattern specific to the COPD and the difference between COPD patients and healthy controls.

In this study, we seek to determine the domains of cognitive function impaired in the COPD patients again and explore the brain areas showing difference in ALFF between groups of COPD patients and healthy controls. We also try to investigate the possible relationship between alteration in ALFF and the affected cognitive function as well as lung function test using correlation analysis. Meanwhile, supplemental oxygen is commonly adopted as an auxiliary therapy at home for COPD patients. However, it still remains to be explored whether home oxygen therapy will have a direct impact on the brain activity pattern in COPD patients. Moreover, considering the impact of both hypoxia and hyperoxia in the spontaneous blood oxygen level‐dependent (BOLD) signal, we attempted to explore whether concurrent oxygen inhalation will affect ALFF in brain areas showing difference between COPD patients and healthy controls (Bulte, 2016). This may help to increase our knowledge of the physiologic origin of ALFF and shed some light on the mechanism of the effect of home oxygen therapy.

## 2 MATERIALS AND METHODS

### 2.1 Participants

This study was approved by the Institutional Ethics Committee of Zhongda Hospital, Medical School of Southeast University (Nanjing, China). Written informed consent was given to all the participants.

A total of 28 stable stage COPD patients and 26 age, sex, and education level matched healthy controls were enrolled in this study from 2014 to 2017. All the patients were undergoing drug therapy at home before and during the study. The drugs include long‐acting beta agonists, inhaled corticosteroids, and tiotropium bromide. Exclusion criteria include patients with acute exacerbation of COPD, patients with dementia, patients with diagnoses of other neuropsychiatric diseases, history of brain surgery, alcohol or drug abuse, history of recent use of drugs affecting cognitive functions (less than 2 months), diabetes patients, patients with obstructive sleep apnea‐hypopnea syndrome and contraindications to MR examinations. All the subjects were enrolled from the local community and have an education level of primary school at least.

### 2.2 Laboratory tests

Spirometry tests without bronchodilator were performed to evaluate the function of pulmonary ventilation on all the participants within 1 week before MRI examination. At the same time, the COPD patients also underwent an arterial blood gas analysis to ensure that they were not hypoxemic or hypercapnic patients. Subjects who could not finish the spirometry tests would not be enrolled for the next MR scan section.

### 2.3 Neuropsychological assessments

Neuropsychological tests including Mini‐Mental State Examination (MMSE) Test, the Montreal Cognitive Assessment (MoCA) Test, Complex Figure Test (CFT), Auditory Verbal Learning Test (AVLT), Digit Span Test (DST), Digit Symbol Substitution Test (DSST), Trail‐Making Test (TMT‐A and TMT‐B) were assessed 2 hours before MRI in all the participants. The CFT has a recognition section and a recall section. The time used for recognition section of the CFT was also recorded.

### 2.4 MR imaging data acquisition

MR imaging data were acquired with a Siemens 3‐T Trio scanner (Erlangen, Germany) at the Radiology Department of Zhongda Hospital. A gradient‐echo planar sequence was used to acquire the BOLD fMRI images using the following parameters: 36 slices; repetition time, 2,000 ms; echo time, 25 ms; slice thickness, 4 mm; flip angle, 90°; field of view, 240 mm × 240 mm; in‐plane resolution, 64 × 64; repetition, 180; Time to Acquisition, 6 min). Structural images were obtained using a 3D T1‐weighted spoiled gradient‐recalled sequence (sagittal; field of view, 256 × 256 mm; in‐plane resolution, 256 × 256; 176 slices; slice thickness, 1 mm; repetition time, 1,900 ms; echo time, 2.48 ms; inversion time, 900 ms; flip angle, 9°; and section gap, 0 mm). Fluid‐attenuated inversion recovery (FLAIR) images were also obtained for evaluation of cerebral vessel disease (repetition time, 8,500 ms; echo time, 94 ms; 20 slices; slice thickness, 5 mm; in‐plane resolution, 256 × 256; voxel size, 0.45 × 0.45 × 5 mm^3^).

To assess the impact of supplementary oxygen using for the ALFF values, a subset of participants including eight COPD patients and eight controls underwent two rs‐fMRI runs. In the first run, all the subjects breathed freely with room air. Before the second run, 100% O_2_ had been delivered with a flow rate of 2.5 L/min (FiO_2_ = 0.31) through a nasal cannula to the participants for about 25 min and continued until the end of the fMRI session. The normal controls were under moderate hyperoxia during the second run.

### 2.5 Cerebral small vessel disease assessment

White matter change (WMC) was assessed on T2WI, T1WI, and FLAIR images with a rating scale described previously (25). Participants with a whole brain rating score >4 (the sum of white matter and basal ganglia rating score) were excluded from the experiment. Two radiologists (with 3‐ and 5‐year experience) blinded to the group allocations performed the ratings separately. Consensus was achieved through consultation to a senior radiologist with 10‐year experience.

### 2.6 Image data processing

All the images were preprocessed based on Statistical Parametric Mapping software (SPM8, http://www.fil.ion.ucl.ac.uk/spm/). Structural images and functional images were preprocessed with the Data Processing Assistant for Resting‐State Functional MR Imaging toolbox (DPARSF; http://www.restfmri.net/forum/DPARSF). The preprocessing steps for functional images included the following: eliminating the first 10 points, slice timing correction, realignment for head motion correction, spatial normalization, spatial smoothness with an isotropic Gaussian kernel (FWHM = 4 mm), and nuisance covariates regression for the drift terms, head motion parameters, CSF signals, and white matter signals. Any subjects with head motion >2.0 mm translation or >2.0° rotation were excluded. The DARTEL method was used for the segmentation of 3D T1 images and normalization of both functional and structural images. A band‐pass filtering (0.01–0.1 Hz) was performed in the BOLD images and the ALFF values of each voxel were calculated using a rs‐fMRI data analysis toolkit (REST1.8, http://www.restfmri.net).

### 2.7 Statistical analysis

A one‐sample *t* test was performed on the normalized ALFF (subtract mean divide by standard deviation, zALFF) images of the COPD group to review the brain areas of the significant higher ALFF (*p* < 0.005, AlphaSim corrected). Voxel‐wise two‐sample *t* tests were performed in both the gray matter volume images and zALFF images between the COPD group and control group, with age, education levels, smoking (pack‐years), and whole brain WMC rating score as covariates. Multiple comparison corrections were performed using family‐wise error rate correction with a cluster defining threshold *p* = 0.001 (two tail), corresponding to a cluster level of *p* = 0.05. The cluster showing difference in the two‐sample *t* test was served as ROI to extract the zALFF value for further analysis.

Except for image data, all the other data were analyzed using The SPSS 18.0 software (SPSS Inc. Chicago, IL). The Student's *t* test and the Mann–Whitney *U* test were used to compare normally distributed and non‐normally distributed group values respectively. The chi‐squared test was used to detect gender significance between groups. Paired *t* test was used to detect the difference of zALFF values before and after oxygen inhalation. The Pearson correlations were utilized to explore the relationship between zALFF values and neuropsychological performance as well as other clinical indexes showing group difference. Multiple correlations were corrected by the FDR (False Discovery Rate) correction.

## 3 RESULTS

### 3.1 Demographic and clinical profiles

Two COPD patients and four healthy controls were excluded because of excessive head motion. The clinical profile of remaining subjects is summarized in Table 1. All the subjects enrolled in this study are male. There were no significant differences in age or years of education between COPD patients and healthy controls. The control group had smoked significantly less than COPD patients regarding both pack‐years and ratio of smokers (*p* = 0.01). In pulmonary function test, the COPD patients performed significantly worse than the healthy controls. The profile of FEV1%pred (predicted % forced expiratory volume in 1 min) indicated a moderate airway obstruction in COPD patients. Moderate hypoxemia (82.55% ± 11.90%) were observed in the COPD patients and mean PaCO_2_ were in the normal range (38.29% ± 5.26%). But the SaO_2_ was significantly lower in COPD patients than in healthy controls. In the cerebral vessel disease assessment, there were more subcortical lesions in COPD patients. The COPD patients had a worse performance in the general cognitive impairment (MMSE and MoCA) tests than the healthy controls. When referring to the specific domains, COPD patients got lower scores in the reverse DST, DSST, TMT‐A, and TMT‐B tests. Meanwhile, patients with COPD spent more time on the recognition section of CFT than healthy controls.

**Table 1:** Clinical profile of COPD patients and controls

	COPD patients (*n* = 26)	Controls (*n* = 22)	*P*‐value
Age (years)	63.12 ± 5.3	61.68 ± 4.63	0.335
Sex (m/f)	26/0	22/0	NA
Years of education	9.73 ± 2.20	10.41 ± 2.26	0.299
Disease duration	9.63 ± 7.81	NA	NA
COPD staging (*n*)		NA	NA
Very mild (FEV_1_%pred ≥ 80)	2	NA	NA
Moderate (FEV_1_%pred 50–79)	7	NA	NA
Severe (FEV_1_%pred 30–49)	9	NA	NA
Very severe (FEV_1_%pred < 30)	8	NA	NA
Smoking (y/n)	22/4	11/11	0.01
Smoking (Pack‐years)	35.20 ± 24.04	14.64 ± 16.93	0.002
SaO_2_ (%)	94.70 ± 3.46	97.64 ± 1.13	<0.001
Spirometry			
FVC%pred	67.34 ± 21.95	100.32 ± 10.55	<0.001
FEV_1_%pred	48.00 ± 25.16	101.24 ± 14.06	<0.001
FEV_1_/FVC (%)	51.11 ± 14.57	79.41 ± 6.34	<0.001
Blood gas analysis			
PaO_2_ (%)	82.55 ± 11.90	NA	NA
PaCO_2_ (%)	38.29 ± 5.26	NA	NA
WMC rating score			
White matter	1 (0–2)	1 (0–2)	0.009
Basal ganglia	0.5 (0–2)	0 (0–2)	0.017
Whole brain	2 (0–4)	1 (0–4)	0.002
Neuropsychological tests			
MMSE	26.15 ± 1.51	27.81 ± 1.37	<0.001
MoCA	21.17 ± 3.86	24.09 ± 2.65	0.004
CFT recognition	34.54 ± 3.22	35.36 ± 1.29	0.267
CFT‐delayed recall (20 min)	13.88 ± 6.05	16.09 ± 7.00	0.247
Task time for CFT recognition	198.35 ± 56.92	153.73 ± 53.97	0.008
AVLT	14.12 ± 3.38	14.45 ± 3.51	0.736
AVLT‐delayed recall (20 min)	7.77 ± 4.75	9.63 ± 3.32	0.114
Forward DST	13.54 ± 2.61	14.00 ± 2.02	0.503
Reverse DST	6.84 ± 1.99	8.23 ± 1.41	0.009
DSST	29.46 ± 9.72	38.31 ± 10.30	0.004
TMT‐A	62.71 ± 18.04	52.50 ± 14.92	0.018
TMT‐B	167.23 ± 49.10	135.40 ± 29.12	0.011

Note

Data were presented as mean ± *SD* or median (range).

Abbreviations: SaO_2_, Oxygen Saturation of Arterial Blood; WMC, White matter change; FVC, Forced Vital Capacity; FEV1, Forced Expiratory Volume in the first second; FVC%pred, ratio of FVC to predicted FVC; FEV_1_%pred, ratio of FEV_1_ to predicted FEV_1_; PaO_2_, Partial Pressure of Oxygen; PaCO_2_, Partial Pressure of Carbon Dioxide; MMSE, Mini‐Mental State Examination; MoCA, Montreal Cognitive Assessment; CFT, Complex Figure Test; AVLT, Auditory Verbal Learning Test; DST, Digit Span Test; DSST, Digit Symbol Substitution Test; TMT‐A, Trail‐making test‐A; TMT‐B, Trail‐making test‐B.

### 3.2 Structural and functional MRI result

No significant difference was observed in regional gray matter (GM) volume or white matter (WM) volume in the voxel‐wise analysis after multiple comparison correction between COPD patients and control group. There was also no significant difference in the total GM volume and total WM volume (Table 2).

**Table 2:** Total gray matter and white matter volume

	COPD	Control	*p*
Total GM volume	0.68 ± 0.048	0.71 ± 0.06	0.15
Total WM volume	0.52 ± 0.04	0.53 ± 0.05	0.42
Brain Parenchyma	1.21 ± 0.09	1.24 ± 0.11	0.25

Note

GM, gray matter; WM, white matter.

**Figure 1:**
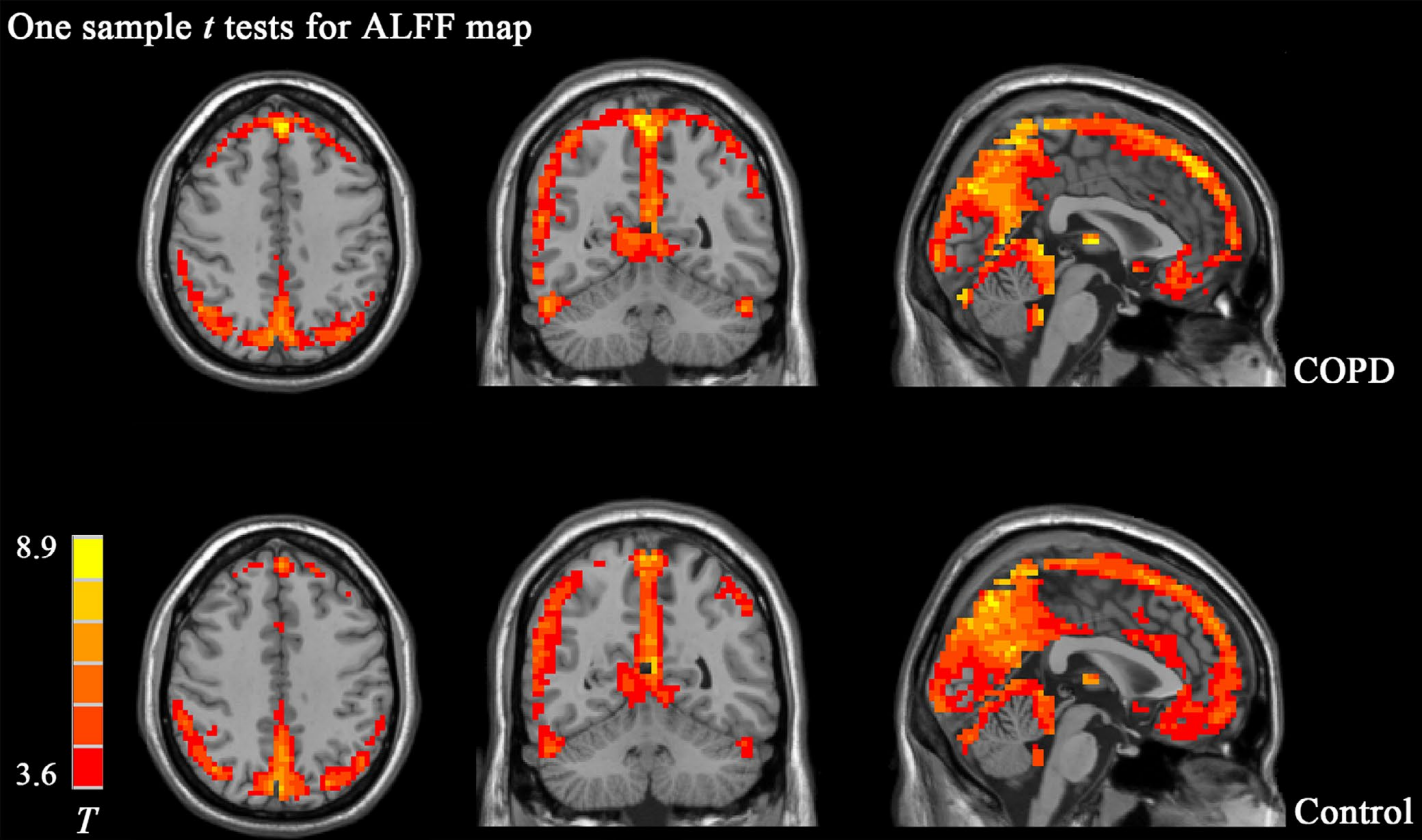
One‐sample *t* test s of ALFF map in COPD group and control group. T maps showing areas (Red color) in which ALFF value is significantly greater than whole brain mean are listed on the upper panel for COPD group, and bottom panel for Control group. Those areas mainly include the posterior cingulate cortex (PCC), precuneus (PCu), and medial prefrontal cortex (mPFC) which resembles the pattern of default‐mode network. No apparent difference was observed in the basic patterns of ALFF between the two groups

**Figure 2:**
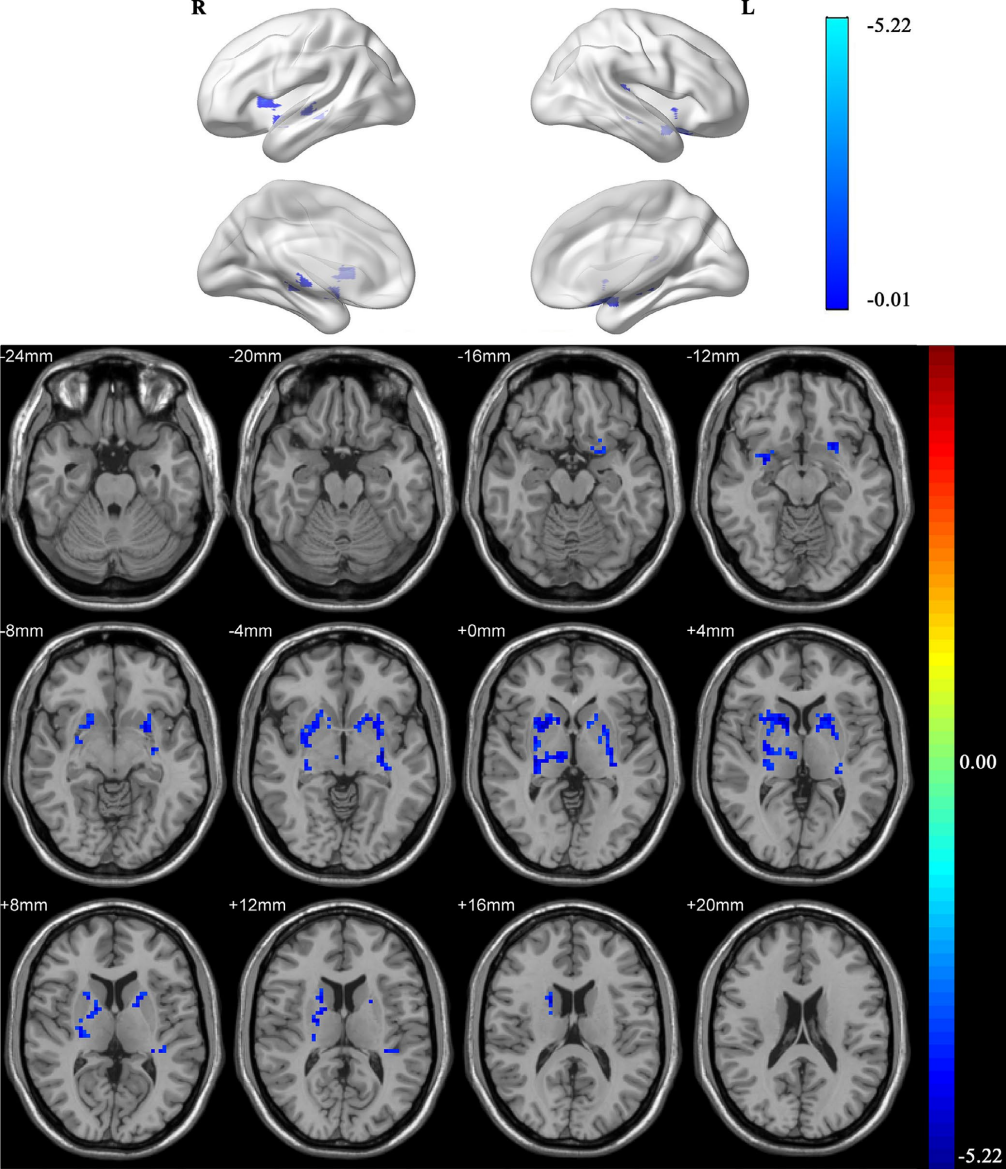
Two‐sample *t* test of ALFF map between COPD group and control group. Decreased ALFF values were mainly located in the bilateral caudate, putamen, pallidum, and thalamus in COPD patients

In one‐sample *t* test of the ALFF map, the posterior cingulate cortex, precuneus, and medial prefrontal cortex demonstrate a significantly higher ALFF value than the global mean in both groups, which resembles the pattern of the default mode network (Figure 1). Compared to healthy control, decreased ALFF values were mainly found in the bilateral basal ganglia areas including caudate, putamen and pallidum in COPD patients (Figure 2). Meanwhile, the right thalamus also exhibited lower ALFF in the COPD patients than that in controls. Detailed information of clusters showing difference in two‐sample *t* test is summarized in Table 3.

**Table 3:** Clusters of two‐sample *t* test of zALFF images between COPD patients and controls

Cluster	Brain areas (AAL)	Voxel Size	Peak Coordinate			Area of peak voxel	*z*‐Peak
			*X*	*Y*	*Z*		
1	R Putamen	90	14	14	3	R Caudate	−5.22
	R Thalamus	27					
	R Caudate	22					
	R Pallidum	10					
	R Other areas (including WM)	87					
2	L Putamen	88	−18	9	6	R Putamen	−5.1
	L Pallidum	10					
	L Caudate	7					
	L Other areas (including WM)	59					

Note

WM, white matter.

### 3.3 Correlation analysis

**Figure 3:**
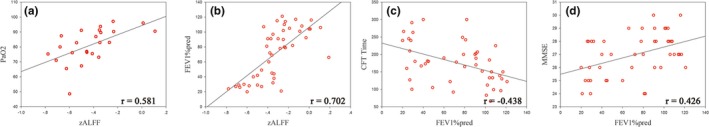
Correlation analysis between aberrant ALFF value and clinical indexes. Aberrant ALFF value correlates with PaO_2_ in COPD group and correlates closely with FEV1%pred in all subjects (a and b, respectively). FEV1%pred correlates with task time for CFT recognition and MMSE as well (c and d, respectively)

ALFF values were extracted from all the subjects using the clusters showing significant difference in the two‐sample *t* test as ROI. The ALFF value correlated closely with the pulmonary function performance and particularly with FEV1%pred (*r* = 0.702, *p* < 0.0001; *r* = 0.672, *p* < 0.0001; and *r* = 0.613, *p* < 0.0001 for FEV1%pred, FEV1/FVC and FVC%pred respectively, Figure 3). The correlation of pulmonary function performance and the ALFF value remains significant when the analysis were performed in the COPD group (*r* = 0.601, *p* = 0.002; *r* = 0.602, *p* = 0.002 for FEV1%pred and FEV1/FVC respectively) but not in the control group. Meanwhile, the ALFF value correlated with PaO_2_ in the COPD group (*r* = 0.581, *p* = 0.003). The ALFF value also correlated with MMSE test (*r* = 0.383, *p* = 0.009) and task time for CFT recognition (*r* = −0.373, *p* = 0.011), although it did not reach significance after correction for multiple comparison. Furthermore, the correlations between ALFF value and PaO_2 _or neuropsychological test performance did not exist after controlling the FEV1%pred. And then, we investigated the relationship between FEV1%pred and neuropsychological test performance (Figure 3) and found that FEV1%pred significantly correlated with MMSE test (*r* = 0.426, *p* = 0.003) and task time for CFT recognition (*r* = −0.438, *p* = 0.002). The correlation remained significant after controlling for the smoking and small cerebral vessel disease (*r* = 0.371, *p* = 0.013; *r* = −0.402, *p* = 0.006 for correlation with MMSE performance and task time for CFT recognition). The correlation coefficient between the ALFF value and FEV1%pred remained significant after controlling PaO_2 _in the COPD group.

### 3.4 Alteration in ALFF after oxygen inhalation

**Figure 4:**
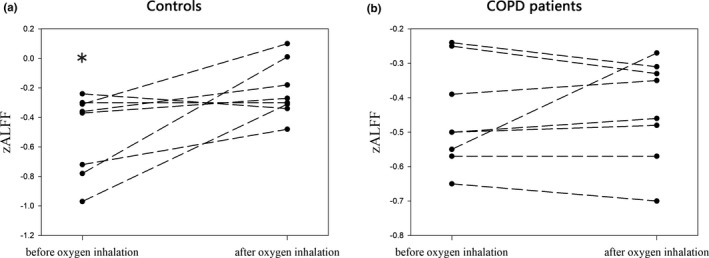
Change in ALFF in deep brain of COPD patients and healthy controls after supplementary oxygen inhalation. After oxygen delivery, the ALFF value of basal ganglia and right thalamus significantly increased in the control group (a), but not in the COPD group (b)

A subset of subjects including eight COPD patients and eight controls underwent a second run with oxygen delivered concurrently through a nasal cannula during MRI session. ALFF values were extracted from all the subjects using the same ROI of correlation analyses and compared between the first run and the second run within each group. After oxygen inhalation, the ALFF value of basal ganglia and right thalamus significantly increased in the control group (*T* = −2.57, *p* = 0.037, Figure 4), but not in the COPD group (*T* = −0.556, *p* = 0.596).

## 4 DISCUSSION

The COPD patients mainly exhibited declines in the executive function, while the function of long‐term memory (Delayed CFT and AVLT) is comparable between the two groups. This result is largely coincided with the finds of Dodd et al. and Cleutjens et al. (Cleutjens, Franssen, et al., 2017; Dodd et al., 2012). The affected domains indicate that the COPD mainly associated with na‐MCI rather than a‐MCI, which is also supported by the results of previous studies (Singh et al., 2014; Villeneuve et al., 2012). Another interesting finding is that it takes longer time for COPD patients to copy a complicated line drawing in the recognition section of the CFT than normal control. This result reminds us of a previous study reporting that drawing ability could predict mortality and was uniquely associated with mortality in severe COPD (Antonelli‐Incalzi et al., 2006).

Our study demonstrates more extensive WMC in the COPD patients compared to normal controls. Impaired white matter microstructural integrity in COPD patients has been reported by many studies (Dodd et al., 2012; Spilling, Jones, Dodd, & Barrick, 2017); van Dijk et al., 2004). However, the results of GM volume change reported by previous studies are inconsistent (Dodd et al., 2012; Esser et al., 2016; Spilling et al., 2017; Wang et al., 2017). Some studies revealed that there existed regional or global brain atrophy in COPD patients (Esser et al., 2016; Wang et al., 2017), while other studies did not find a significant difference in GM volume between COPD patients and normal controls (Dodd et al., 2012; Spilling et al., 2017). In our study, the COPD patients did not demonstrate a significant different GM or WM volume when compared to normal controls. In our opinion, severe hypoxemia will finally lead to brain atrophy as reflected by the chronic phase of hypoxic‐ischemic brain injury (Heinz & Rollnik, 2015; Weiss, Galanaud, Carpentier, Naccache, & Puybasset, 2007), while brain atrophy may not be prevalent in the stable COPD patients. Meanwhile, a rigid multiple comparison correction method adopted in our statistical tests may also account for the negative result (Eklund, Nichols, & Knutsson, 2016).

The decreased ALFF of BOLD signal in deep brain could be related to altered neurovascular coupling and suppressed spontaneous neuronal activity under chronic hypoxia environment. One study demonstrated that the magnitude of task‐related BOLD response varied with the region of the brain under hypoxia (Dunn, Wadghiri, & Meyerand, 1999). In our study, the basal ganglia nuclei were most sensitive to mild hypoxia regarding ALFF measurement, which is due to its anatomical position. However, it is also important to know that lower PaO_2_ implies lower oxyhemoglobin and higher deoxyhemoglobin in the blood, which lead to a lower baseline signal intensity in the T2‐star weighed BOLD‐fMRI images. The time cost for CFT recognition is associated with motor function and executive function (Royall, 2006). It is well‐accepted that the damage of basal ganglia is responsible for the motor function impairment in the basal ganglia stroke patients. Moreover, basal ganglia is also involved in the executive function including working memory and decision‐making (Helie, Ell, & Ashby, 2015; Schroll & Hamker, 2013). Furthermore, there exists a significant correlation between ALFF signal and PaO_2_. Thus, there is a possibility that decreased ALFF value in the bilateral basal ganglia may directly owing to the lower PaO_2 _and may be associated with the suppressed neuronal activity leading to motor and/or executive function impairment. Also, impairment of pulmonary ventilation function may be the original cause of the fMRI findings and CI because of the highest correlation coefficient between FEV1%pred and other aberrant pathophysiology indexes.

Oxygen therapy is a common treatment for COPD patients with moderate to severe hypoxemia. Short‐term oxygen inhalation increased the PaO_2_ in the COPD patients and leads to moderate hyperoxia in normal controls (Prieur et al., 1998). There are some studies reporting elevated BOLD signal intensity in rats and humans under hyperoxia (Losert, Peller, Schneider, & Reiser, 2002; Sicard & Duong, 2005), and elevated baseline BOLD signal increases the ALFF value mathematically. The unchanged ALFF signal in the deep brain of COPD patients should be interpreted with caution. There have been an adaptive change of neurons in deep brain nuclei under long‐term mild hypoxia, which leads to the amplitude of such low‐frequency (0.01–0.1) BOLD signal fluctuation remain unchanged. Alternatively, it is also possible that ALFF does not change so sensitively to the increase in PaO_2 _in a relatively lower range in COPD patients. A recent study claims that the cerebral vasculature of COPD patients is insensitive to low‐flow oxygen delivery because of the unaltered CBF (Hoiland et al., 2018). There are some reports indicating that long‐term oxygen treatment will not improve the survival of COPD patients with moderate resting hypoxemia (Chaouat et al., 1999; Gorecka, Gorzelak, Sliwinski, Tobiasz, & Zielinski, 1997). It will be interesting to investigate whether the unchanged ALFF value in deep brain nuclei of COPD patients could be related to these results.

There is a limitation that we did not evaluate the possible neurovascular coupling change of the COPD patients so that we could not reach a conclusion about the exact underlying mechanism of the altered ALFF in the deep brain of these patients. However, a recent report revealed unaltered neurovascular coupling under moderate hypoxia (Decroix et al., 2018). Another limitation of this study is that the sample size is too small for the oxygen inhalation data analysis, which may not be able to reveal the real effect of oxygen inhalation in COPD patients. Finally, interpretation of the relationship between altered ALFF and other clinical indexes is difficult since most of the correlations were nonsignificant in separate groups. Notably, some old COPD patients showed difficulty in focusing on cognitive tasks for a long time, which limit the accuracy of the neuropsychological tests. This can also ultimately lead to a relatively lower correlation coefficient between altered ALFF and scores of the tests.

In conclusion, this study supports the association between COPD and na‐MCI and highlights the executive function impairment in COPD patients. Our study also revealed the aberrant ALFF alteration in the deep brain of these patients, and this alteration may be directly related to the PaO_2_ changes and play a role in the CI of these COPD patients.

## CONFLICT OF INTEREST

The authors declare no potential conflict of interests.

## DATA AVAILABILITY STATEMENT

The data that support the findings of this study are available on request from the corresponding author. The data are not publicly available due to privacy or ethical restrictions.

## References

[brb31336-bib-0001] Bulte, D. (2016). Hyperoxia and functional MRI. Advances in Experimental Medicine and Biology, 903, 187–199. 10.1007/978-1-4899-7678-9_13 27343097

[brb31336-bib-0002] Chang, S. S. , Chen, S. , McAvay, G. J. , & Tinetti, M. E. (2012). Effect of coexisting chronic obstructive pulmonary disease and cognitive impairment on health outcomes in older adults. Journal of the American Geriatrics Society, 60(10), 1839–1846. 10.1111/j.1532-5415.2012.04171.x 23035917 PMC3470752

[brb31336-bib-0003] Chaouat, A. , Weitzenblum, E. , Kessler, R. , Charpentier, C. , Ehrhart, M. , Schott, R. , … Moutinho dos Santos, J. (1999). A randomized trial of nocturnal oxygen therapy in chronic obstructive pulmonary disease patients. European Respiratory Journal, 14(5), 1002–1008. 10.1183/09031936.99.14510029 10596681

[brb31336-bib-0004] Cleutjens, F. A. , Franssen, F. M. , Spruit, M. A. , Vanfleteren, L. E. , Gijsen, C. , Dijkstra, J. B. , … Janssen, D. J. (2017). Domain‐specific cognitive impairment in patients with COPD and control subjects. International Journal of Chronic Obstructive Pulmonary Disease, 12, 1–11. 10.2147/COPD.S119633 28031706 PMC5182042

[brb31336-bib-0005] Cleutjens, F. A. H. M. , Ponds, R. W. H. M. , Spruit, M. A. , Burgmans, S. , Jacobs, H. I. L. , Gronenschild, E. H. B. M. , … Janssen, D. J. A. (2017). The relationship between cerebral small vessel disease, hippocampal volume and cognitive functioning in patients with COPD: An MRI study. Frontiers in Aging Neuroscience, 9, 88 10.3389/fnagi.2017.00088 28424613 PMC5371656

[brb31336-bib-0006] Cleutjens, F. A. H. M. , Spruit, M. A. , Ponds, R. W. H. M. , Vanfleteren, L. E. G. W. , Franssen, F. M. E. , Dijkstra, J. B. , … Janssen, D. J. A. (2017). The impact of cognitive impairment on efficacy of pulmonary rehabilitation in patients with COPD. Journal of the American Medical Directors Association, 18(5), 420–426. 10.1016/j.jamda.2016.11.016 28108209

[brb31336-bib-0007] Cui, Y. , Jiao, Y. , Chen, Y.‐C. , Wang, K. , Gao, B. , Wen, S. , … Teng, G.‐J. (2014). Altered spontaneous brain activity in type 2 diabetes: A resting‐state functional MRI study. Diabetes, 63(2), 749–760. 10.2337/db13-0519 24353185

[brb31336-bib-0008] Decroix, L. , De Pauw, K. , Van Cutsem, J. , Pattyn, N. , Heyman, E. , & Meeusen, R. (2018). Acute cocoa flavanols intake improves cerebral hemodynamics while maintaining brain activity and cognitive performance in moderate hypoxia. Psychopharmacology (Berl), 235(9), 2597–2608. 10.1007/s00213-018-4952-2 29951768

[brb31336-bib-0009] Dodd, J. W. , Chung, A. W. , van den Broek, M. D. , Barrick, T. R. , Charlton, R. A. , & Jones, P. W. (2012). Brain structure and function in chronic obstructive pulmonary disease: A multimodal cranial magnetic resonance imaging study. American Journal of Respiratory and Critical Care Medicine, 186(3), 240–245. 10.1164/rccm.201202-0355OC 22652026

[brb31336-bib-0010] Dodd, J. W. , Getov, S. V. , & Jones, P. W. (2010). Cognitive function in COPD. European Respiratory Journal, 35(4), 913–922. 10.1183/09031936.00125109 20356988

[brb31336-bib-0011] Dunn, J. F. , Wadghiri, Y. Z. , & Meyerand, M. E. (1999). Regional heterogeneity in the brain's response to hypoxia measured using BOLD MR imaging. Magnetic Resonance in Medicine, 41(4), 850–854. 10.1002/(SICI)1522-2594(199904)41:4<850:AID-MRM27>3.0.CO;2-L 10332864

[brb31336-bib-0012] Eisner, M. D. , Iribarren, C. , Blanc, P. D. , Yelin, E. H. , Ackerson, L. , Byl, N. , … Katz, P. P. (2011). Development of disability in chronic obstructive pulmonary disease: Beyond lung function. Thorax, 66(2), 108–114. 10.1136/thx.2010.137661 21047868 PMC3111223

[brb31336-bib-0013] Eklund, A. , Nichols, T. E. , & Knutsson, H. (2016). Cluster failure: Why fMRI inferences for spatial extent have inflated false‐positive rates. Proceedings of the National Academy of Sciences of the USA, 113(28), 7900–7905. 10.1073/pnas.1602413113 27357684 PMC4948312

[brb31336-bib-0014] Esser, R. W. , Stoeckel, M. C. , Kirsten, A. , Watz, H. , Taube, K. , Lehmann, K. , … von Leupoldt, A. (2016). Structural brain changes in patients with COPD. Chest, 149(2), 426–434. 10.1378/chest.15-0027 26203911

[brb31336-bib-0015] Fried, T. R. , Vaz Fragoso, C. A. , & Rabow, M. W. (2012). Caring for the older person with chronic obstructive pulmonary disease. JAMA, 308(12), 1254–1263. 10.1001/jama.2012.12422 23011715 PMC3815613

[brb31336-bib-0016] Gavello, D. , Rojo‐Ruiz, J. , Marcantoni, A. , Franchino, C. , Carbone, E. , & Carabelli, V. (2012). Leptin counteracts the hypoxia‐induced inhibition of spontaneously firing hippocampal neurons: A microelectrode array study. PLoS ONE, 7(7), e41530 10.1371/journal.pone.0041530 22848520 PMC3405131

[brb31336-bib-0017] Goodall, S. , Twomey, R. , & Amann, M. (2014). Acute and chronic hypoxia: Implications for cerebral function and exercise tolerance. Fatigue, 2(2), 73–92. 10.1080/21641846.2014.909963 25593787 PMC4292893

[brb31336-bib-0018] Gorecka, D. , Gorzelak, K. , Sliwinski, P. , Tobiasz, M. , & Zielinski, J. (1997). Effect of long‐term oxygen therapy on survival in patients with chronic obstructive pulmonary disease with moderate hypoxaemia. Thorax, 52(8), 674–679. 10.1136/thx.52.8.674 9337824 PMC1758616

[brb31336-bib-0019] Heinz, U. E. , & Rollnik, J. D. (2015). Outcome and prognosis of hypoxic brain damage patients undergoing neurological early rehabilitation. BMC Research Notes, 8, 243 10.1186/s13104-015-1175-z 26081628 PMC4469251

[brb31336-bib-0020] Helie, S. , Ell, S. W. , & Ashby, F. G. (2015). Learning robust cortico‐cortical associations with the basal ganglia: An integrative review. Cortex, 64, 123–135. 10.1016/j.cortex.2014.10.011 25461713

[brb31336-bib-0021] Hoiland, R. L. , Mladinov, S. , Barak, O. F. , Willie, C. K. , Mijacika, T. , Stembridge, M. , … Ainslie, P. N. (2018). Oxygen therapy improves cerebral oxygen delivery and neurovascular function in hypoxaemic chronic obstructive pulmonary disease patients. Experimental Physiology, 103(8), 1170–1177. 10.1113/EP086994 29978513

[brb31336-bib-0022] Lahousse, L. , Tiemeier, H. , Ikram, M. A. , & Brusselle, G. G. (2015). Chronic obstructive pulmonary disease and cerebrovascular disease: A comprehensive review. Respiratory Medicine, 109(11), 1371–1380. 10.1016/j.rmed.2015.07.014 26342840

[brb31336-bib-0023] Losert, C. , Peller, M. , Schneider, P. , & Reiser, M. (2002). Oxygen‐enhanced MRI of the brain. Magnetic Resonance in Medicine, 48(2), 271–277. 10.1002/mrm.10215 12210935

[brb31336-bib-0024] Prieur, F. , Busso, T. , Castells, J. , Bonnefoy, R. , Benoit, H. , Geyssant, A. , & Denis, C. (1998). Validity of oxygen uptake measurements during exercise under moderate hyperoxia. Medicine and Science in Sports and Exercise, 30(6), 958–962. 9624658 10.1097/00005768-199806000-00028

[brb31336-bib-0025] Raffaele, A.‐I. , Andrea, C. , Claudio, P. , Luigi, T. , Domenico, A. , Aldo, S. , … Franco, R. (2006). Drawing impairment predicts mortality in severe COPD. Chest, 130(6), 1687–1694. 10.1378/chest.130.6.1687 17166983

[brb31336-bib-0026] Royall, D. R. (2006). Double jeopardy. Chest, 130(6), 1636–1638. 10.1378/chest.130.6.1636 17166974

[brb31336-bib-0027] Schroll, H. , & Hamker, F. H. (2013). Computational models of basal‐ganglia pathway functions: Focus on functional neuroanatomy. Frontiers in Systems Neuroscience, 7, 122 10.3389/fnsys.2013.00122 24416002 PMC3874581

[brb31336-bib-0028] Sicard, K. M. , & Duong, T. Q. (2005). Effects of hypoxia, hyperoxia, and hypercapnia on baseline and stimulus‐evoked BOLD, CBF, and CMRO2 in spontaneously breathing animals. NeuroImage, 25(3), 850–858. 10.1016/j.neuroimage.2004.12.010 15808985 PMC2962945

[brb31336-bib-0029] Singh, B. , Mielke, M. M. , Parsaik, A. K. , Cha, R. H. , Roberts, R. O. , Scanlon, P. D. , … Petersen, R. C. (2014). A prospective study of chronic obstructive pulmonary disease and the risk for mild cognitive impairment. JAMA Neurology, 71(5), 581–588. 10.1001/jamaneurol.2014.94 24637951 PMC4020948

[brb31336-bib-0030] Singh, B. , Parsaik, A. K. , Mielke, M. M. , Roberts, R. O. , Scanlon, P. D. , Geda, Y. E. , … Petersen, R. C. (2013). Chronic obstructive pulmonary disease and association with mild cognitive impairment: The Mayo clinic study of aging. Mayo Clinic Proceedings, 88(11), 1222–1230. 10.1016/j.mayocp.2013.08.012 24182702 PMC3875365

[brb31336-bib-0031] Spilling, C. A. , Jones, P. W. , Dodd, J. W. , & Barrick, T. R. (2017). White matter lesions characterise brain involvement in moderate to severe chronic obstructive pulmonary disease, but cerebral atrophy does not. BMC Pulm Med, 17(1), 92 10.1186/s12890-017-0435-1 28629404 PMC5474872

[brb31336-bib-0032] Sulaiman, I. , Cushen, B. , Greene, G. , Seheult, J. , Seow, D. , Rawat, F. , … Costello, R. W. (2017). Objective assessment of adherence to inhalers by patients with chronic obstructive pulmonary disease. American Journal of Respiratory and Critical Care Medicine, 195(10), 1333–1343. 10.1164/rccm.201604-0733OC 27409253

[brb31336-bib-0033] Sumiyoshi, A. , Suzuki, H. , Shimokawa, H. , & Kawashima, R. (2012). Neurovascular uncoupling under mild hypoxic hypoxia: An EEG‐fMRI study in rats. Journal of Cerebral Blood Flow and Metabolism, 32(10), 1853–1858. 10.1038/jcbfm.2012.111 22828997 PMC3463877

[brb31336-bib-0034] van Dijk, E. J. , Vermeer, S. E. , de Groot, J. C. , van de Minkelis, J. , Prins, N. D. , Oudkerk, M. , … Breteler, M. M. (2004). Arterial oxygen saturation, COPD, and cerebral small vessel disease. Journal of Neurology, Neurosurgery and Psychiatry, 75(5), 733–736. 10.1136/jnnp.2003.022012 PMC1763550 15090569

[brb31336-bib-0035] Villeneuve, S. , Pepin, V. , Rahayel, S. , Bertrand, J.‐A. , de Lorimier, M. , Rizk, A. , … Gagnon, J.‐F. (2012). Mild cognitive impairment in moderate to severe COPD: A preliminary study. Chest, 142(6), 1516–1523. 10.1378/chest.11-3035 23364388

[brb31336-bib-0036] Wang, C. , Ding, Y. , Shen, B. , Gao, D. , An, J. , Peng, K. , … Qiu, S. (2017). Altered gray matter volume in stable chronic obstructive pulmonary disease with subclinical cognitive impairment: An exploratory study. Neurotoxicity Research, 31(4), 453–463. 10.1007/s12640-016-9690-9 28005183

[brb31336-bib-0037] Weiss, N. , Galanaud, D. , Carpentier, A. , Naccache, L. , & Puybasset, L. (2007). Clinical review: Prognostic value of magnetic resonance imaging in acute brain injury and coma. Critical Care, 11(5), 230 10.1186/cc6107 17980050 PMC2556735

[brb31336-bib-0038] Yu‐Feng, Z. , Yong, H. E. , Chao‐Zhe, Z. , Qing‐Jiu, C. , Man‐Qiu, S. , Meng, L. , … Yu‐Feng, W. (2007). Altered baseline brain activity in children with ADHD revealed by resting‐state functional MRI. Brain and Development, 29(2), 83–91. 10.1016/j.braindev.2006.07.002 16919409

